# Suggestions Regarding a Laboratory of Neurology and Psychology at the University of Pennsylvania

**Published:** 1899-01

**Authors:** Charles K. Mills

**Affiliations:** Philadelphia, Pa., Professor of mental diseases and of medical jurisprudence in the University of Pennsylvania


### Selections.

### SUGGESTIONS REGARDING A LABORATORY OF NEUROLOGY AND PSYCHOLOGY AT THE UNIVERSITY OF PENNSYLVANIA.

By CHARLES K. MILLS, M. D., Philadelphia, Pa.,

Professor of mental diseases and of medical jurisprudence in the University of Pennsylvania.

OF THE subjects set apart as specialties by universal consent, neurology—under which general designation psychiatry should be included—is one of the broadest. In investigation and in literature more advances have been made in recent years in this than in other of the special branches of medicine. Far-reaching discoveries in psychology, and in the anatomy and physiology of the nervous system have been developed. Much of the work has been done abroad, although during recent years, some zealous and energetic American workers have successfully entered the field. The methods of neurological teaching have made comparatively little advance in this country; and to prepare for research, teaching and practical work at home, the American student seeks the well-equipped neurological and psychological laboratories of Europe.

One of the special objects of this communication is to call attention to some of these deficiences, to make suggestions as to their remedy, and to appeal to those interested to assist in putting on a firm foundation the teaching of this subject at the University of Pennsylvania.

It is not the intention of the writer to criticise the university authorities or any of its teachers. The conditions which exist are due largely to lack of clinical and laboratory facilities, which has necessitated the perpetuation of methods not fully abreast of the times; and it is our hope that, if attention is clearly called to this need, the friends of advanced medical education, and especially those interested in psychology and neurology, will come forward with material assistance.

Although the methods of neurology at the University of Pennsylvania are as good, if not better, than those followed in nearly all the American medical schools, they are but little better than those which prevailed twenty years ago. One clinical lecture a week is delivered by the clinical professor of nervous diseases in the hospital of the university, this instruction being supplemented by some practical work in nervous diseases and electrotherapeutics by assistants. In addition, the writer gives during four months of the regular medical course a weekly clinical lecture on either mental or nervous diseases to those students of the fourth year who elect such instruction. The material of these lectures is drawn from the neurological department of the Philadelphia hospital, in the arena of which institution the lectures are delivered. The professor of the practice of medicine and of the practice of clinical medicine also gave limited attention to nervous diseases. Neurology, so far as examinations are concerned, is ranked among the "major electives" of the fourth year, the students being examined for their standing in this elective by the clinical professor of nervous diseases. Practically all neurological courses at the university are optional.

The results attained by this imperfect method of teaching a subject so important are sometimes shown in the examination for internes by the hospitals of Philadelphia. The student will learn to respect the subject and to pursue it with diligence when it is made one of the compulsory branches, one on which he must pass a satisfactory examination before receiving his diploma. The neurological instruction offered to students of the university should not be limited to that furnished by one or even by two chairs.

If the medical department of the university had in official connection with it a hospital for the insane, or well-equipped and well-endowed wards for patients suffering from mental diseases, it might be well to have the chair of psychiatry entirely separate from that of neurology (excluding psychiatry); but under the conditions it is probably best, as at the present, to have two clinical professors, each teaching both psychiatry and general neurology. In any case the teacher of psychiatry should be classed with the other professors, having the same recognition as is accorded to others holding chairs of equal importance.

By extramural professors and lecturers the abundant and valuable neurological material of various hospitals in the City of Philadelphia could easily be made accessible to those university students who are especially interested in neurology.

A hospital or hospital wards for nervous and mental diseases should be organically united with laboratories of research. It is only by supplementing careful work in hospital and private practice, by laboratory research and teaching that the best results in the diagnosis and treatment of diseases of the nervous system can be achieved. Why should not the alumni and friends of the university, and those interested in maintaining the preëminence of Philadelphia as a medical center, lend their assistance to this end by contributing the funds to erect on the grounds of the university a building which, I would suggest, might well be made a testimonial to Dr. S. Weir Mitchell, by designating it the Weir Mitchell Laboratory of Neurology and Psychology?

The name of Weir Mitchell holds the first place in the neurological history of this country. As physician, investigator and author he is one whom all should desire to honor. It is fit that a recognition of his position and services, recalling the field of medicine in which he has so successfuly labored, should be made in connection with the University of Pennsylvania, to the development of which he has so largely contributed.

Should the project be realised, it might be best to combine under one roof, or in connected buildings, opportunities both for research and for teaching, so that to advanced undergraduates and to post-graduates instruction in experimental psychology, and in the anatomy and pathology of the nervous system, could be offered either in independent courses or in connection with clinical neurology, while special researches could at the same time be pursued in rooms set apart and properly equipped.

The neurological laboratory should contain models, diagrams, and gross preparations of the brain, spinal cord, peripheral nerves and sense-organs; mounted specimens of both normal and abnormal tissues; and the preserving fluids, reagents, jars, microtomes, microscopes and other appliances necessary both for research and for teaching. It is impossible to gain any accurate knowledge of nervous pathology, except by the actual study of the specimens. In the teaching of students, and even in research, such study is too often divorced from clinical investigation. In this laboratory would be gradually accumulated an invaluable collection representing the pathological anatomy of nervous diseases.

In connection with this laboratory should be a photographic room, with the necessary appliances for taking photographs of cases which appear at the out-door and in-door services of the hospital. Much material, valuable in illustrating neurological lectures and contributions, is lost for lack of such facilities.

With a laboratory of psychology closely connected with such a neurological laboratory and with hospital wards, psychometric and reaction-time researches, studies in sense phenomena, and in memory, and psycho-physiological investigations in general, could be made on the diseased as well as on the healthy.

From such laboratories would go forth publications containing the results of much original investigation.

The director of such a laboratory, or of its neurological subdivision, should be professor of neuropathology, and for the more efficient development of the subject should also be one of the physicians to the neurological wards.

It may be said that other matters of greater importance await completion before the energies and the material aid of the friends of the university should be directed to the development of a special subject or branch of instruction like neurology; but experience has shown that often several movements of a somewhat similar character can be carried forward together with almost as much ease as one. A specially announced purpose in the development of the University of Pennsylvania during the ensuing year is the erection and endowment of new laboratories. It is to be a "laboratory year" preëminently and why should not a neurological and psychological laboratory, which has so much to commend it in the ends of the medical department, be included in the list of the important additions soon to be made to the university?

If four years, the time now embraced in the full course of instruction in the University of Pennsylvania, as in other medical schools of equal rank, is not sufficient to carry out methods of instruction such as would be indicated by the suggestions as to the teaching of neurology in this communication, a course of five years should be speedily established, but it may not be absolutely necessary to do this at present if the curriculum was thoroughly organised.—*University Medical Magazine*, November, 1898.

